# RDoC cognitive systems and emerging psychopathology: A latent variable analysis of teacher-reported psychosocial difficulties and executive function processes in young children

**DOI:** 10.1017/S003329172610508X

**Published:** 2026-07-07

**Authors:** Steve Eaton, Stephanie H. M. van Goozen

**Affiliations:** 1 School of Psychology, Cardiff University, Cardiff, UK; 2 Department of Clinical Neurodevelopmental Studies, Leiden University, Leiden, the Netherlands

**Keywords:** executive functioning, P-Factor, psychopathology, RDoC, transdiagnostic, cognitive systems, structural equation modelling (SEM), latent variable, strengths and difficulties questionnaire (SDQ), early intervention

## Abstract

**Background:**

Executive function (EF) deficits are consistently linked to psychopathology symptoms, though the mechanisms linking poor EF to symptom expression remain unclear.

**Methods:**

The study used the Research Domain Criteria (RDoC) approach to examine relationships between teacher-reported latent psychopathology symptoms, including a general psychopathology factor (P-Factor), and EF in young children with emerging mental health problems. Participants were 804 children (70.8% male; aged 49–89 months) referred by their teachers for cognitive, emotional, or behavioral problems at school. To assess psychopathology, teachers completed the Strengths and Difficulties Questionnaire (SDQ). EF measures included inhibition, cognitive flexibility, working memory, sustained attention, and episodic memory, assessed using the NIH Toolbox, Automated Working Memory Assessment, and the Amsterdam Neuropsychological Test battery.

**Results:**

Structural equation modeling (incorporating confirmatory factor analysis) showed reasonable model fit and supported a P-Factor structure. Correlational analyses explored EF–psychopathology associations, followed by a sensitivity analysis controlling for sex. We observed patterns of cognitive processes that showed inverse associations between EF performance and specific clinical problems. Sustained attention was positively associated with emotional problems but negatively associated with hyperactivity problems. Sex-stratified analyses revealed distinct patterns, with inhibition problems strongly linked to conduct and hyperactivity problems, but in females only.

**Conclusions:**

The findings support poor EF as a transdiagnostic risk factor associated with incremental vulnerability for childhood psychopathology. Divergent findings for sustained attentional processes suggest they can be adaptive in some contexts but maladaptive in others. Screening for EF difficulties in children could enhance early identification and inform interventions.

## Introduction

Early detection of emotional and behavioral difficulties in childhood is essential for reducing long-term psychiatric risk, yet the mechanisms that underlie this vulnerability remain poorly understood (Askelund et al., 2023). Emotional and behavioral problems can be broadly categorized into internalizing and externalizing dimensions. Internalizing symptoms are characterized by internal distress and include disorders such as anxiety and depression, whereas externalizing symptoms are defined by outwardly directed behaviors and include disorders such as attention-deficit hyperactivity disorder (ADHD) and conduct disorder (Achenbach, McConaughy, & Howell, 1987).

Internalizing/externalizing dimensions provide a framework with which to understand the shared and distinct characteristics of emerging psychiatric symptoms. A growing body of research suggests that these broadband dimensions and the behaviors that comprise them can be collapsed into a single dimension that reflects a general liability for psychopathology (P-Factor; Patalay et al., 2015; Ortuño-Sierra et al., 2015). Conceptualized as a transdiagnostic risk factor, the P-Factor captures shared variance across internalizing and externalizing dimensions, offering a dimensional approach to understanding psychiatric risk (Caspi et al., 2014).

The National Institute of Mental Health’s Research Domain Criteria (RDoC) supports a dimensional perspective and emphasizes transdiagnostic approaches to investigate the neurobiological and cognitive processes that give rise to psychopathology. The RDoC approach, therefore, moves beyond traditional diagnostic systems, such as the Diagnostic and Statistical Manual of Mental Disorders (DSM; APA, 2022), that categorize mental disorders into discrete conditions. While categorical classification can be clinically useful, it nevertheless obscures the dimensional nature of psychiatric symptoms (from mild to severe) and fails to capture common symptoms that cut across diagnostic boundaries. The RDoC approach highlights the importance of dimensional, transdiagnostic processes, such as executive functioning (EF), in understanding the development and maintenance of psychopathology (Insel et al., 2010).

### Core executive functions and neurodevelopmental disorders

Considered essential processes for goal-directed behaviors and self-regulation, EF falls under the cognitive systems domain of the RDoC matrix. Core EFs consist of three separable but related processes: working memory (WM), inhibition, and flexibility (Diamond, 2013; Miyake et al., 2000). Compared to typically developing peers, children with internalizing and externalizing symptoms can demonstrate impairments in EF (Yang et al., 2022). For example, relative weakness in inhibitory control and working memory is linked with externalizing behaviors such as ADHD (Anning, Langley, Hobson, & Van Goozen, 2023; Burley, Anning, & Van Goozen, 2022), conduct disorder (Deters et al., 2020), and oppositional defiant disorder (Deters et al., 2020; Schoorl et al., 2018). Similarly, difficulties in inhibition, working memory, and flexibility have been associated with internalizing behaviors such as depression and anxiety (Murphy et al., 2018; Snyder, 2013). Low flexibility/rigidity has also been proposed as being characteristic of autistic spectrum disorder (ASD; D’Cruz et al., 2013); however, the evidence for this is inconsistent (Geurts, Corbett, & Solomon, 2009).

Higher-order processes, such as sustained attention and episodic memory, are shaped and constrained by core EFs. Intact attention relies on inhibition (to block irrelevant stimuli), flexibility (to adapt to new information), and working memory (to keep task goals in mind). Impairments in these EFs manifest as impulsiveness, distractibility, and hyperactivity – a cluster of behaviors prominent in ADHD (Friedman et al., 2007). Episodic memory is similarly scaffolded by the core EFs, relying on inhibition (for optimal coding and retrieval), working memory (to maintain elements of an episode), and flexibility (for strategic retrieval), and impairments in these processes are linked to poor episodic memory in children with ADHD (Anning et al., 2023) and heightened ASD symptoms (Geng et al., 2024). Lower episodic memory performance also hinders the ability to recall and learn from social experiences, contributing to problems with social communication (McQuade, Murray-Close, Shoulberg, & Hoza, 2013; Picard et al., 2012).

Taken together, these findings suggest that difficulties in core and higher-order EFs support the view that it is one of several interacting transdiagnostic processes that explain risk for a broad range of psychopathology, although the specific EF patterns can vary depending on disorder and symptom type.

### Child psychopathology ratings

Parents usually initiate assessments for potential psychopathology in their children, and so are typically the primary informant source. However, parents can be motivated to over- or underreport symptoms. Parental reports can also be subjective, reflect emotional biases such as denial or overprotection, and some parents have limited exposure to other children, meaning they can struggle with determining age-appropriate behavior (Achenbach et al., 1987). In contrast, teachers observe a larger number of children of a similar age in structured settings in which psychosocial and behavioral demands are consistent, meaning they have a normative benchmark for development. This makes them well-placed to identify behaviors that are unusual or problematic in contexts other than home.

The present study integrates the above perspectives by examining associations between latent psychopathology dimensions, including a higher-order P-Factor, derived from teacher-reported behavioral measures, and assessments of EF in young children who are displaying emerging mental health problems at school. In line with the RDoC framework, this approach moves beyond the perspective of categorical diagnoses to identify early transdiagnostic, dimensional processes. In turn, these processes could inform intervention strategies applicable to multiple forms of childhood maladjustment.

Our hypotheses were informed by previous literature and findings from our own lab, and these were that (i) core EFs (inhibition, flexibility, and working memory) would be inversely associated with the general psychopathology factor; (ii) inhibition, sustained attention, and episodic memory would be inversely associated with externalizing problems (operationalized by the conduct and hyperactivity subscales); and (iii) working memory and flexibility would be inversely associated with internalizing problems (operationalized by the emotional and peer problems subscales).

## Methods

### Participants

Participants were 804 children (70.8% male; 29.2% female) aged 49–89 months (*M* = 76.40, SD = 12.32). Children were referred to the Neurodevelopment Assessment Unit (NDAU; https://www.cardiff.ac.uk/neurodevelopment-assessment-unit) by their teachers for exhibiting emerging and significant emotional or behavioral difficulties in the classroom. None had a diagnosis of a neurodevelopmental disorder. The study was performed in accordance with ethical standards described in the 1964 Declaration of Helsinki. Written informed consent was obtained from the participants’ parent or caregiver, and all procedures were approved by the Cardiff University Research Ethics Committee (EC.16.10.11.4592GR).

### Measures

#### Psychopathology

Emerging psychopathology was assessed using teacher-reported scores from the Strengths and Difficulties questionnaire (SDQ; Goodman, 1997). We focused on four ‘Difficulties’ subscales: emotional problems, conduct problems, hyperactivity problems, and peer problems. Each scale can be categorized into bands, which identify problem severity – ‘Close to Average’, ‘Slightly raised’, ‘High’, or ‘Very high’, with those scoring ‘High’ or ‘Very high’ indicating a need for clinical intervention.

The hyperactivity/inattention subscale of the SDQ is strongly associated with ADHD symptoms (Stone et al., 2010), the emotional problems subscale with internalizing difficulties such as anxiety and depression (Muris, Meesters, & Van den Berg, 2003), the conduct problems subscale with conduct disorder (Goodman et al., 2000), and the peer problems subscale with social difficulties (Goodman, 2001). As such, each subscale of the SDQ has been shown to effectively map onto established clinical constructs, providing a reliable index of emerging psychiatric disorders.

Structural equation modeling (SEM), incorporating confirmatory factor analysis (CFA), was used to derive latent constructs for each domain as well as a higher-order general psychopathology factor (P-Factor), and to assess model fit. Items with factor loadings ≤ .60 were excluded from the model to ensure robust representation of the latent variables (Field, 2005).

#### Cognitive systems measures

The NIH Toolbox (Akshoomoff et al., 2014) was used to assess cognitive inhibition, flexibility, and episodic memory. Tests were administered on a tablet computer. Age-corrected scores were used in the analysis, which have a mean of 100 and a standard deviation of 15.

Cognitive inhibition was measured using the Flanker task. Participants were asked to select a button that matches a target stimulus while suppressing their attention to the flanking stimuli. Congruent trials showed an arrow pointing in the same direction as the flanking stimuli; incongruent trials showed an arrow pointing in the opposite direction from the flanking stimuli. Scores reflect response time and accuracy, corrected for age.

Cognitive flexibility was measured using the Dimensional Change Card Sorting (DCCS) task. Participants are presented with an image and required to match it to one of two possible responses at the bottom of their screen, based on either shape or color. Scores reflect response time and accuracy, corrected for age.

Episodic memory was measured using the Picture Sequence Memory (PSM) task. Participants were required to recall the order in which a series of images describing the steps of an activity was presented. The number of images increases with each trial, for a maximum of three trials. Scores are derived from using the cumulative number of adjacent pairs of images correctly recalled. Raw scores are converted to a theta score using item response theory.

Working memory (WM) was assessed using the Automated Working Memory Assessment (AWMA; Alloway, Gathercole, Kirkwood, & Elliott, 2008). In this task, participants are given strings of spoken digits and tasked to recall them in reverse order. The strings increase in number as the task progresses.

Sustained attention was measured using the ‘Pursuit’ task from the Amsterdam Neuropsychological Test battery (ANT; De Sonneville, 1999). Participants are required to track a randomly moving target as closely as possible on a computer screen for 5 minutes. The distance of the cursor from the target in the first and last 30 s is measured, with negative scores indicating lower and positive scores indicating higher sustained attention.

### Procedure

Teachers completed the SDQ as part of the child’s referral to NDAU. EF tasks were administered individually to children by trained graduate researchers. Standardized instructions were given for all tasks.

### Data analysis

SEM models were created using IBM SPSS AMOS Version 29. All other statistical analyses were performed using SPSS Version 29.

First, the latent structure of the SDQ domains and the higher-order P-Factor were confirmed. Model fit was assessed using absolute fit indices (*χ* ^2^), comparative fit indices (Comparative Fit Index (CFI), Tucker–Lewis Index (TLI), and error of approximation indices (root mean square error of approximation [RMSEA]). Next, correlational analyses were run between EF measures and both domain-specific and general psychopathology factors.

## Results

Descriptive information for age, teacher-rated SDQ scores, latent variables, and executive functioning measures for the whole sample, and by sex, is presented in Table 1.

**Table 1.** Descriptive statistics (*N* = 804) Table 1. long description.

					Test statistics (males vs. females)		
	Mean (SD)	Range	Skewness	Kurtosis	*T*	*p*	% High/Very high, SDQ
Sex	70.8%M/29.2%F						
Age (in months)	76.40 (12.32)	49–100	−0.09	−0.83	−1.15*	.05	
Teacher rated SDQ scores							
Emotional problems	3.19 (2.67)	0–10	0.59	−0.65	−3.95**	<.001	30.6%
Conduct problems	3.43 (2.66)	0–10	0.43	−0.80	3.53**	<.001	51.7%
Hyperactivity problems	7.64 (2.61)	0–10	−1.06	0.31	5.26**	<.001	71.3%
Peer problems	3.43 (2.28)	0–10	0.13	−0.93	1.69	.09	36.2%
Total problems	17.71 (6.55)	0–36	−0.23	−0.42	2.48*	.01	68.2%
Latent variables							
Emotion	0 (.51)	−.63–1.12	.52	−.88	−3.42**	<.001	
Conduct	0 (.35)	−.46–.73	.55	−.93	4.69**	<.001	
Hyperactivity	0 (.36)	−.77–.31	−.92	−.59	4.73**	<.001	
Peer	0 (.29)	−.42–.62	.14	−1.12	1.67	.09	
P-Factor	0 (.94)	−2.28 – 2.49	−.12	−.39	2.15*	.03	
Executive functioning measures							
Working memory (AWMA)	97.02 (16.48)	61–141	0.02	−0.36	1.04	.30	
Episodic memory (PSM)	99.14 (18.60)	51–146	0.51	−0.14	−0.15	.88	
Flexibility (DCCS)	93.34 (14.08)	54–132	−0.25	−0.25	−2.00*	.05	
Inhibition (Flanker)	90.37 (14.07)	52–131	−0.18	0.02	1.02	.31	
Sustained attention (Pursuit)	−17.31 (31.11)	−175.11 – 84.79	1.53	2.88	−0.88	.38	

Note: *p ≤ .05; **p ≤ .001.

Independent *t*-tests revealed significant sex differences across several domains. Females were significantly older than males, exhibited higher levels of emotional problems, and outperformed males in cognitive flexibility. Conversely, males showed higher conduct, hyperactivity, and general psychopathology (P-Factor) scores. No significant sex differences were found for peer problems or for any of the other executive functioning measures. Due to the presence of some sex differences, sensitivity analyses were performed, consisting of repeating the analyses when (i) controlling for sex, and (ii) with males and females stratified into separate subgroups. Due to the age-corrected and standardized results across many of the EF tasks, age was not controlled for in further analysis.

### Model fit

Table 2 presents model fit statistics. Because the *χ* ^2^ test is sensitive to large sample sizes, the *p*-value for this test was likely to be significant in our sample. Therefore, the absolute fit indices are reported here as a convention only (Goodboy & Kline, 2017). Overall, model fit indices suggested a reasonable fit (CFI = 0.89; RMSEA = 0.07).

**Table 2.** Model fit summary Table 2. long description.

Chi-square minimum model	NPAR	CMIN	DF	*P*	CMIN/DF
Default model	53	572.86	101	.000	5.79
Saturated model	152	.000	0		
Independence model	32	4558.10	120	.000	37.98

Baseline comparisons model	NFI	RFI	IFI	TLI	CFI
	Delta1	rho1	Delta2	rho2	
Default model	0.87	0.85	0.89	0.87	0.89
Saturated model	1		1		1
Independence model	0	0	0	0	0

Root mean square error of approximation model (RMSEA)					
Model	RMSEA	LO 90	HI 90	PCLOSE	
Default model	0.07	0.06	0.08	.000	
Independence model	0.22	0.21	0.22	.000	

### Factor loadings

Confirmatory factor analysis supported the latent structure of the ‘Difficulties’ subdomains of the SDQ. Supplementary Table S1 shows factor loadings for each item, and the loading of each subdomain (peer, emotional, conduct, and hyperactivity problems) on the P-Factor. Although only 30.6% of the sample fell within the ‘high’ or ‘very high’ range for emotional problems, this domain showed the strongest loading on the P-Factor (61%). This reflects the fact that the factor loadings capture shared variance across the distribution of scores, rather than the prevalence of those cases that crossed an arbitrary threshold. Overall, factor loadings were moderate to strong across all domains, with emotional problems showing the greatest contribution to the P-Factor, followed by hyperactivity (41%), conduct (38%), and peer problems (30%).

Supplementary Table S2 displays factor score weights for each item. Each item yielded a weight that was high in its subdomain only and low on all other factors, suggesting that the items discriminated well between constructs. Four items were found to have factor loadings lower than 0.60 and were therefore removed from the analysis. Responses to these items were mostly zero (‘Not true’; Supplementary Table S3). The low response frequencies for these items likely reflect behaviors that were either outside of the teacher’s awareness (e.g. sickness) or that they were unlikely to acknowledge as happening (e.g. theft in the classroom).

### Dimensional associations between variables

Supplementary Table S4 shows correlation coefficients between the latent variables. All subdomains were strongly correlated with the P-Factor. Additionally, the severity of peer problems was positively correlated with all other problem domains. Because conduct and hyperactivity are part of the externalizing domain, and peer problems are part of the internalizing domain, this finding was not expected. However, because we used teacher ratings, it is reasonable to assume that conduct problems influence the quality and frequency of a child’s social interactions at school, similar to how hyperactivity problems could be associated with peer rejection due to socially inappropriate behaviors (McQuade et al., 2013). All four latent domains were found to be strongly and positively associated with the P-Factor.

Measures of executive functioning were assessed for correlation strength to check for evidence of multicollinearity (Supplementary Table S5). None of the coefficients suggested multicollinearity was present across the measures (threshold = 0.80; Tabachnick & Fidell, 2019). Working memory, episodic memory, flexibility, and inhibition were positively correlated, while sustained attention showed negative associations with the other EF tasks, as expected.

Table 3 provides correlation coefficients between latent psychopathology variables and measures of executive functioning. Broadly, the observed inverse associations between EF and latent psychopathology symptoms suggested that better EF performance was generally linked to fewer problems. Specifically, negative associations were found between working memory and peer problems (*r* = −.08), episodic memory and conduct problems (*r* = −.11), flexibility and peer (*r* = −.16) and hyperactivity problems (*r* = −.08), and inhibition and hyperactivity (*r* = −.11). Both flexibility and inhibition were negatively associated with the P-factor (*r* = −.11; *r* = −.09). Sustained attention showed mixed results, as it was positively associated with emotional problems (*r* = .10) but negatively associated with hyperactivity (*r* = −.10).

**Table 3.** Associations between latent variables and EF measures Table 3. long description.

	Working memory (AWMA)		Episodic memory (PSM)		Flexibility (DCCS)		Inhibition (Flanker)		Sustained attention (Pursuit)	
	*r*	*P*	*r*	*P*	*r*	*P*	*r*	*P*	*r*	*P*
Latent variable										
Emotional problems	.02	.54	.03	.38	−.01	.70	−.01	.76	.10*	.01
Peer problems	−.08*	.04	−.05	.20	−.16^**^	<.001	−.05	.18	.01	.85
Conduct problems	−.06	.10	−.11*	.004	−.05	.18	−.07	.09	.05	.18
Hyperactivity problems	−.07	.08	−.03	.40	−.08*	.05	−.11*	.01	−.10*	.03
P-Factor	−.06	.11	−.05	.19	−.11*	.01	−.09*	.02	.003	.95

Note: * = p ≤ .05; ** = p ≤ .001.

### Sensitivity analyses

Because significant sex differences were observed across some latent variables, relationships between these variables and EF performance were analyzed when holding sex constant (Supplementary Table S6), and then separately, in males (Supplementary Table S7) and females (Supplementary Table S8). When sex was held constant, the results were broadly consistent with the main findings, although emotional problems were no longer positively associated with sustained attention, and flexibility was no longer negatively associated with hyperactivity problems or the P-Factor. In males only, no latent variables were significantly associated with sustained attention or inhibition, and flexibility was no longer negatively associated with hyperactivity problems. In females, no latent variables were significantly associated with working memory or flexibility, but episodic memory was now significantly associated with peer problems and not with conduct problems. Furthermore, inhibition was now associated with conduct problems. Relationships between latent variables and EF performances were observed with stronger effect sizes in females despite the smaller sample size, which reduced power. These findings should be interpreted cautiously however, they suggest that sex-specific profiles exist in the association between EF and emerging psychopathology.

## Discussion

The present study used the RDoC framework to examine associations between teacher-reported psychopathology, indexed both at domain-specific and general (P-Factor) levels by the Strengths and Difficulties Questionnaire (SDQ) and different executive functioning (EF) measures in a large sample of young children referred for an assessment by their teachers due to emerging psychosocial problems exhibited at school.

Structural equation modeling (SEM) supported the notion of a higher-order P-Factor structure (Caspi et al., 2014), showing that the P-Factor dimension explained variance across domains. ‘Emotional problems’ most strongly contributed to the P-Factor (61%), suggesting that internalizing symptoms such as anxiety may be a core dimension of general psychopathology in young children. The weaker factor loading for peer problems may reflect the contextual nature of peer difficulties, which are potentially both shaped and limited by teacher perceptions.

The observed associations between EF performance and latent psychopathology were modest in magnitude. Although effects of this size are common in developmental psychopathology, particularly for transdiagnostic and dimensional constructs (Schäfer & Schwarz, 2019; Weinerová, Szűcs, & Ioannidis, 2022), they should be interpreted as a reflection of modest associations and not strong causal effects.

### Executive function and psychopathology symptoms through the RDoC framework

Associations between EF and psychopathology were modest but consistent and highlight cognitive control mechanisms in children that are associated with emerging internalizing and externalizing difficulties. Our first prediction, that core EFs (inhibition, flexibility, and working memory) would be negatively associated with the P-Factor, was not fully met. That is, we found that poorer inhibition and flexibility – but not working memory (WM) – were inversely associated with the P-Factor, suggesting that these processes are particularly relevant to general psychopathology risk. This finding supports the role of inhibition and flexibility as core mechanisms of cognitive control and self-regulation (Diamond, 2013). The lack of association between WM and other core EFs was somewhat surprising, in that core EFs are considered interdependent, and so difficulties in all, or none, were expected. However, WM has been shown to be susceptible to potential moderating factors such as internalizing symptoms or stress brought on by the task itself (Hood et al., 2015).

Our second prediction that inhibition, sustained attention, and episodic memory would be negatively associated with latent hyperactivity was also not fully met. Instead, flexibility, inhibition, and sustained attention – but not episodic memory – were negatively associated with hyperactivity problems. The negative association between flexibility and hyperactivity suggests that flexibility supports top-down attentional control (Farrant, Fletcher, & Maybery, 2014) and contributes to an inconsistent literature, with some studies reporting impaired flexibility in children with ADHD symptoms (Marzocchi et al., 2008) and others not (Geurts et al., 2004). That we did not find associations between episodic memory and hyperactivity appears to be incongruous with other work from our lab (Anning et al., 2023). However, Anning and colleagues used the Child Behavior Checklist (CBCL; Achenbach, Dumenci, & Rescorla, 2003) as a measure of ADHD, which is parent-reported and focuses more on attention problems, reducing the measure’s ability to capture classroom hyperactivity.

Our third prediction that WM and flexibility would be inversely associated with peer problems was met, suggesting that better WM and flexibility were found in those who have healthy relationships with their peers. This illustrates that cognitive control processes support adaptive social functioning in children, possibly through allowing those with intact or superior WM and flexibility to hold, track, and consider multiple perspectives at once, thus improving social interactions (McQuade et al., 2013).

Other findings unrelated to our hypotheses were that episodic memory was negatively associated with conduct problems, suggesting that memory processes can play a protective role against disruptive or harmful patterns of behavior. This could reflect the use of prior experiences to guide present and future behaviors, highlighting episodic memory as a key cognitive system for regulating rule-governed behavior. Neurobiological studies (e.g. Fairchild et al., 2011) have found that, relative to typically developing controls, dorsomedial prefrontal cortex volume can be smaller in those with conduct disorder, and in line with our findings, this area has also been implicated in episodic memory retrieval (Gilbert et al., 2006).

Finally, sustained attention was positively associated with emotional problems but negatively associated with hyperactivity. Though the latter effect was to be expected, the former could be explained by heightened attention in children with elevated internalizing symptoms as they preferentially attend toward threat-related or ruminative content, increasing internalizing symptoms (Eaton, Dorrans, & van Goozen, 2024). These seemingly divergent findings are consistent with RDoC’s goal of exploring cognitive variability. That is, attention can reduce impulsivity (adaptive) but also boost rumination (maladaptive), suggesting that interventions that target cognitive control could benefit children across a broad range of symptom profiles, not just those who meet categorical diagnostic criteria.

Overall, with regard to the RDoC cognitive systems domain, EF may be associated with general psychopathology by constraining children’s ability to regulate attention, behavior, and their emotions across contexts. However, given the developmental stage of the sample, EF should not be interpreted as a causal substrate of the P-Factor. Instead, EF should be considered an associated process that covaries with symptom burden during early development.

### Sex differences

Our sex-stratified analyses revealed that in males, psychopathology was not associated with sustained attention or inhibition, and hyperactivity problems were not associated with flexibility. In females, psychopathology was not associated with working memory or flexibility, and in contrast to the main analysis, episodic memory was negatively associated with peer problems but not with conduct problems. These findings suggest that while similar cognitive systems underlie risk across males and females, their specific manifestations may differ. However, the exploratory nature of these analyses, coupled with limited power in the female subsample, precludes strong inferences. Elsewhere, some reviews (e.g. Grissom & Reyes, 2019) have reported negligible sex differences in EF, whereas others (e.g. Gaillard et al., 2021) find evidence that they are more meaningfully present. Rather than supporting sex-specific interventions, such inconsistency highlights the need for future studies to be specifically designed and powered to consider both shared and sex-specific mechanisms when examining associations between EF and emerging developmental psychopathology.

### Practical implications

It should be noted that our sample was young, meaning that abilities, behaviors, and traits are still emerging, and referred, which means that the observed patterns reflect the sample itself and not true developmental norms. Nevertheless, for children with emerging neurodevelopmental problems, our results emphasize the value of EF as an intervention target for emerging psychiatric problems in children. Classroom practices that scaffold working memory (e.g. breaking tasks into smaller steps), promote flexibility (e.g. encouraging perspective-taking) and bolster inhibition (e.g. using structured routines) can serve as effective interventions that improve functioning and alleviate psychopathology symptoms.

Our findings also suggest that screening for relative problems in EF, alongside behavioral problems, may facilitate the early identification of children at risk for psychopathology. Such screening avoids the pitfalls of narrow symptom-cluster assessments and instead focuses on cognitive processes that drive risk across multiple domains. Sex-stratified interventions may enhance effectiveness.

### Strengths and limitations

The study benefited from a unique and large sample of young children referred by their teachers for demonstrating emerging mental health problems at school. Importantly, children in the sample encompassed a range of difficulties and not exclusively severe symptoms, enhancing real-world and ecological validity. Additionally, the study linked EF to both domain-specific difficulties and a general psychopathology factor, which supports RDoC’s central proposition that common cognitive mechanisms are linked to risk for psychopathology across diagnostic boundaries. The use of latent variables carries with it several strengths relative to summed score approaches, in that summed scores assume that all items contribute equally, which potentially oversimplifies the constructs. Instead, a latent variable approach accounts for unequal item contributions and captures underlying constructs more reliably. Also, SEM allows for the accuracy of the models to be assessed through model fit, whereas using summed data from observed variables lacks the methods necessary to judge whether the scores adequately represent a construct.

The use of teachers as informants can be seen as both a strength and a limitation. Teacher-informed psychopathology ratings benefit from ecologically valid, context-rich observations that can help to identify at-risk individuals earlier. However, although teachers tend to be more adept at noticing externalizing symptoms, parents tend to be more attuned to identifying internalizing symptoms (Murray et al., 2021). As such, the absence of parent reports limits conclusions regarding cross-context generalizability. Using both parent and teacher reports in future work could improve diagnostic validity, helping to capture a full range of symptoms.

In terms of limitations, the cross-sectional design means that causation cannot be inferred. EF difficulties could drive externalizing/internalizing behaviors, or those behaviors stem from EF difficulties, or the relationship is bidirectional. Recent longitudinal evidence (Rungsattatharm, Tasingha, Trairatvorakul, & Chonchaiya, 2025) points to EF difficulties manifesting before the emergence of psychopathology symptoms; however, other research (Zhou et al., 2025) suggests that the relationship is bidirectional, and that while EF ability can protect against internalizing and externalizing problems in early childhood, their symptoms can also influence EF development. Also, although the P-Factor derived from the SDQ demonstrated acceptable model fit, the indices were below thresholds typically considered strong for higher-order latent models. Additionally, the removal of items with low loadings (≤.60) may have affected the content validity of the SDQ domains. The relatively stronger relationship between emotional problems and the P-Factor suggests that it could be weighted more toward internalizing symptoms and should therefore be interpreted as an approximation of general psychopathology rather than an underlying vulnerability.

Also, the study used single measures of each EF process. Multiple measures of EF are preferable, using composite scores from several tasks that assess the same construct (Snyder et al., 2015). Moreover, many of the observed correlations were weak to modest in strength; however, this could be expected because EF tasks capture specific processes under controlled conditions, and latent variables reflect broad behavioral tendencies in a classroom context. Importantly, RDoC emphasizes that even modest associations are meaningful when they reflect underlying cognitive control systems that cut across symptom domains (Insel et al., 2010). Thus, while we accept that the observed relationships are small in magnitude, they nevertheless align with a dimensional, mechanism-focused perspective that advances understanding of early risk for psychopathology using data derived from laboratory and real-world settings.

### Future directions

Future research should adopt longitudinal designs to disentangle causal relationships between EF and emerging psychopathology. Additionally, to enhance measurement precision, future work should employ multiple measures for each EF construct to reduce task-specific variance. Finally, integrating multi-informant perspectives would facilitate comparisons between home and school contexts, in turn accounting for situation-specific behavior.

### Conclusion

The present study points to the value of dimensional, transdiagnostic approaches to increase our understanding of the cognitive basis of emerging psychopathology in young children. Using the SDQ to derive domain-specific latent variables and a general psychopathology factor, we linked these constructs to performances on a range of EF measures and found patterns of cognitive processes associated with specific domains of mental health difficulty. We also found inhibition and flexibility to be negatively associated with the P-Factor. Furthermore, our stratified analysis of sex differences suggests that sex should be considered in terms of future research and interventions, as they may require adjustments to account for potentially distinct profiles of EF difficulties. Collectively, our findings emphasize the importance of targeting EF processes in early prevention and intervention strategies aimed at reducing risk for a broad range of psychopathology.

## Supporting information

10.1017/S003329172610508X.sm001 Eaton and Van Goozen supplementary material Eaton and Van Goozen supplementary material

## Supplementary material

The supplementary material for this article can be found at http://doi.org/10.1017/S003329172610508X.

## Long descriptions

### Table 1. Long description

The table presents data for N = 804 participants. Columns include Mean (S D), Range, Skewness, Kurtosis, T-statistic, p-value, and percentage of High/Very high S D Q scores.

Demographics:

- Sex: 70.8% Male, 29.2% Female.

- Age: Mean 76.40 months (S D 12.32), range 49 to 100.

Teacher rated S D Q scores (Mean, S D, p-value):

- Emotional problems: 3.19 (2.67), p < .001.

- Conduct problems: 3.43 (2.66), p < .001.

- Hyperactivity problems: 7.64 (2.61), p < .001.

- Peer problems: 3.43 (2.28), p = .09.

- Total problems: 17.71 (6.55), p = .01.

Latent variables (Mean, S D, p-value):

- Emotion: 0 (.51), p < .001.

- Conduct: 0 (.35), p < .001.

- Hyperactivity: 0 (.36), p < .001.

- Peer: 0 (.29), p = .09.

- P-Factor: 0 (.94), p = .03.

Executive functioning measures (Mean, S D, p-value):

- Working memory (A W M A): 97.02 (16.48), p = .30.

- Episodic memory (P S M): Not listed with specific values in this row but categorized under executive functioning.

Navigate back to Table 1..

### Table 2. Long description

The table is divided into three horizontal sections.

1. Chi-square minimum model section:

- Default model: N P A R 53, C M I N 572.86, D F 101, P .000, C M I N forward slash D F 5.79.

- Saturated model: N P A R 152, C M I N .000, D F 0.

- Independence model: N P A R 32, C M I N 4558.10, D F 120, P .000, C M I N forward slash D F 37.98.

2. Baseline comparisons model section:

- Default model: N F I (Delta 1) 0.87, R F I (rho 1) 0.85, I F I (Delta 2) 0.89, T L I (rho 2) 0.87, C F I 0.89.

- Saturated model: N F I 1, I F I 1, C F I 1.

- Independence model: N F I 0, R F I 0, I F I 0, T L I 0, C F I 0.

3. Root mean square error of approximation (R M S E A) model section:

- Default model: R M S E A 0.07, L O 90 0.06, H I 90 0.08, P C L O S E .000.

- Independence model: R M S E A 0.22, L O 90 0.21, H I 90 0.22, P C L O S E .000.

Navigate back to Table 2..

### Table 3. Long description

A table with 11 columns. The first column lists Latent variables, followed by pairs of r and p columns for five E F measures: Working memory A W M A, Episodic memory P S M, Flexibility D C C S, Inhibition Flanker, and Sustained attention Pursuit.

* Emotional problems: r values range from minus .01 to .10. Significant association with Sustained attention r equals .10, p equals .01.

* Peer problems: Significant negative associations with Working memory r equals minus .08, p equals .04 and Flexibility r equals minus .16, p is less than .001.

* Conduct problems: Significant negative association with Episodic memory r equals minus .11, p equals .004.

* Hyperactivity problems: Significant negative associations with Flexibility r equals minus .08, p equals .05, Inhibition r equals minus .11, p equals .01, and Sustained attention r equals minus .10, p equals .03.

* P-Factor: Significant negative associations with Flexibility r equals minus .11, p equals .01 and Inhibition r equals minus .09, p equals .02.

Note: single asterisk indicates p is less than or equal to .05; double asterisk indicates p is less than or equal to .001.

Navigate back to Table 3..
