# Supplementary material for: RDoC cognitive systems and emerging psychopathology: A latent variable analysis of teacher-reported psychosocial difficulties and executive function processes in young children
Source: Psychol Med. 2026 Jul 7;56:e222. doi: 10.1017/S003329172610508X (PMC13370183; doi:10.1017/S003329172610508X)
Supplement: Eaton and Van Goozen supplementary material [file S003329172610508Xsup001.docx]

*Supplementary Materials*

*Table S1 – First (item) level and Second (P-Factor) level factor loadings*

| Item | Standardised Regression Weight |
| --- | --- |
| Peer |  |
| SDQ6 - Rather solitary, likes to play alone | .57 |
| SDQ11 - Has at least one good friend | .90 |
| SDQ14 - Generally liked by other children | .57 |
| SDQ23 - Gets on better with adults than with other children | .38 |
| Peer < P-Factor | .30 |
| Emotional |  |
| SDQ8 - Many worries, often seems worried | .77 |
| SDQ13 - Often unhappy, down-hearted or tearful | .57 |
| SDQ16 - Nervous or clingy in new situations, easily loses confidence | .70 |
| SDQ24 - Many fears, easily scared | .77 |
| Emotional < P-Factor | .61 |
| Conduct |  |
| SDQ5 - Often has temper tantrums or hot tempers | .59 |
| SDQ7 - Generally obedient, does what adults request | .75 |
| SDQ12 - Often fights with other children or bullies them | .64 |
| SDQ 18 - Often lies or cheats | .48 |
| Conduct < P-Factor | .38 |
| Hyperactivity |  |
| SDQ2 - Restless, overactive, cannot stay still for long | .92 |
| SDQ10 - Constantly fidgeting or squirming | .85 |
| SDQ15 - Easily distracted, concentration wanders | .61 |
| SDQ25 - Sees tasks through to the end, good attention span | .42 |
| Hyperactivity < P-Factor | .41 |

*Table S2 – Factor Score Weights for each Item*

|  | Peer | Emotional | Conduct | Hyperactivity | P-factor |
| --- | --- | --- | --- | --- | --- |
| Peer |  |  |  |  |  |
| SDQ6 - Rather solitary, likes to play alone | 0.05 | 0.001 | 0.002 | 0.00 | 0.05 |
| SDQ11 (reversed) - Has at least one good friend | 0.25 | 0.01 | -0.01 | 0.001 | 0.26 |
| SDQ14 (reversed) - Generally liked by other children | 0.05 | 0.003 | 0.13 | -0.02 | 0.17 |
| SDQ23 - Gets on better with adults than other children | 0.03 | 0.001 | 0.001 | 0.00 | 0.03 |
| Emotional |  |  |  |  |  |
| SDQ8 - Many worries, often seems worried | 0.006 | 0.26 | -0.001 | 0 | 0.26 |
| SDQ13 - Often unhappy, downhearted or tearful | 0.003 | 0.14 | 0 | 0 | 0.14 |
| SDQ16 - Nervous or clingy in new situations, loses confidence | 0.004 | 0.18 | 0 | 0 | 0.19 |
| SDQ24 - Many fears, easily scared | 0.005 | 0.24 | 0 | 0 | 0.25 |
| Conduct |  |  |  |  |  |
| SDQ7 (reversed) - Generally obedient, does what adults request | 0.004 | -0.002 | 0.17 | 0.01 | 0.18 |
| SDQ12 - Often fights with other children or bullies them | -0.025 | -0.001 | 0.08 | 0.02 | 0.08 |
| SDQ18 - Often lies or cheats | -0.018 | -0.001 | 0.06 | 0.02 | 0.05 |
| SDQ22 - Steals from home, school or elsewhere | -0.002 | 0 | 0.01 | 0.002 | 0.01 |
| Hyperactivity |  |  |  |  |  |
| SDQ2 - Restless, overactive, cannot stay still for long | 0.01 | 0 | 0.02 | 0.52 | 0.55 |
| SDQ10 - Constantly fidgeting or squirming | 0.01 | 0 | 0.01 | 0.32 | 0.34 |
| SDQ15 - Easily distracted, concentration wanders | 0.002 | 0 | 0.004 | 0.10 | 0.11 |
| SDQ25 – Sees tasks through to the end, good attention span | 0.00 | 0.00 | 0.002 | 0.01 | 0.01 |

*Table S3 – Responses to SDQ Items*

|  | Response (*n*/%) | | |
| --- | --- | --- | --- |
|  | 0 = "Not at all" | 1 = "Somewhat True" | 2 = "Certainly True" |
| Peer |  |  |  |
| SDQ6 - Rather solitary, likes to play alone | 291 / 36.2% | 319 / 39.7% | 194 / 24.1% |
| SDQ11 - Has at least one good friend | 261 / 32.5% | 239 / 29.7% | 304 / 37.8% |
| SDQ14 - Generally liked by other children (reversed) | 309 / 38.4% | 383 / 47.6% | 112 / 13.9% |
| SDQ19 - Picked on or bullied by other children | 700 / 87.1% | 92 / 11.4% | 12 / 1.5% |
| SDQ23 - Gets on better with adults than with other children | 371 / 46.1% | 294 / 36.6% | 139 / 17.3% |
| Emotional |  |  |  |
| SDQ3 - Often complains of headaches, stomach aches, or sickness | 603 / 75% | 134 / 16.7% | 67 / 8.3% |
| SDQ8 - Many worries, often seems worried | 343 / 42.7% | 299 / 37.2% | 162 / 20.1% |
| SDQ13 - Often unhappy, down-hearted or tearful | 424 / 52.7% | 262 / 32.6% | 118 / 14.7% |
| SDQ16 - Nervous or clingy in new situations, easily loses confidence | 316 / 39.3% | 261 / 32.5% | 227 / 28.2% |
| SDQ24 - Many fears, easily scared | 455 / 56.6% | 226 / 28% | 124 / 15.4% |
| Conduct |  |  |  |
| SDQ5 - Often has temper tantrums or hot tempers | 350 / 43.5% | 184 / 22.9% | 270 / 33.6% |
| SDQ7 - Generally obedient, does what adults request (reversed) | 218 / 27.1% | 329 / 40.9% | 257 / 32% |
| SDQ12 - Often fights with other children or bullies them | 465 / 57.8% | 179 / 22.3% | 160 / 19.9% |
| SDQ 18 - Often lies or cheats | 453 / 56.3% | 221 / 27.5% | 130 / 16.2% |
| SDQ22 - Steals from home, school or elsewhere | 654 / 81.3% | 92 / 11.4% | 58 / 7.2% |
| Hyperactivity |  |  |  |
| SDQ2 - Restless, overactive, cannot stay still for long | 134 / 16.7% | 181 / 22.5% | 489 / 60.8% |
| SDQ10 - Constantly fidgeting or squirming | 145 / 18% | 203 / 25.2% | 456 / 56.7% |
| SDQ15 - Easily distracted, concentration wanders | 65 / 8.1% | 146 / 18.2% | 593 / 73.8% |
| SDQ21 - Thinks things out before acting (reversed) | 65 / 8.1% | 113 / 24% | 626 / 77.9% |
| SDQ25 - Sees tasks through to the end, good attention span (reversed) | 74 / 9.2% | 196 / 24.4% | 534 / 66.4% |

*Table S4 – Correlations between latent variables*

|  | Emotional Problems | | Conduct Problems | | Hyperactivity Problems | | Peer Problems | |
| --- | --- | --- | --- | --- | --- | --- | --- | --- |
|  | r | p | r | p | r | p | r | p |
| Conduct Problems | .01 | .70 | -- |  |  |  |  |  |
| Hyperactivity Problems | -.04 | .21 | .49^**^ | <.001 | -- |  |  |  |
| Peer Problems | .30^**^ | <.001 | .20^**^ | <.001 | .17^**^ | <.001 | -- |  |
| P-Factor | .63^**^ | <.001 | .63^**^ | <.001 | .60^**^ | <.001 | .61^**^ | <.001 |

*Note: ** = p≤.001*

*Table S5 – Correlations between executive functions*

|  | Working Memory (AWMA) | | Episodic Memory (PSM) | | Flexibility (DCCS) | | Inhibition (Flanker) | |
| --- | --- | --- | --- | --- | --- | --- | --- | --- |
|  | r | p | r | p | r | p | r | p |
| Episodic Memory (PSM) | .33^**^ | <.001 | -- |  |  |  |  |  |
| Flexibility (DCCS) | .27^**^ | <.001 | .18^**^ | <.001 | -- |  |  |  |
| Inhibition (Flanker) | .33^**^ | <.001 | .23^**^ | <.001 | .36^**^ | <.001 | -- |  |
| Sustained Attention (Pursuit) | -.13^**^ | 0.001 | -.09^*^ | 0.03 | -0.02 | 0.67 | -.16^**^ | <.001 |

*Note: * = p≤.05; ** = p≤.001*

*Table S6 – Associations between latent variables and EF measures (sex held constant)*

|  | Working Memory (AWMA) | | Episodic Memory (PSM) | | Flexibility (DCCS) | | Inhibition (Flanker) | | Sustained Attention (Pursuit) | |
| --- | --- | --- | --- | --- | --- | --- | --- | --- | --- | --- |
|  | *r* | *p* | *r* | *p* | *r* | *p* | *r* | *p* | *r* | *p* |
| Latent Variable |  |  |  |  |  |  |  |  |  |  |
| Emotional problems | .04 | .34 | .03 | .44 | -.01 | .81 | -.01 | .71 | -.07 | .11 |
| Peer problems | -.09* | .03 | -.003 | .94 | -.11* | .01 | -.05 | .25 | -.02 | .64 |
| Conduct problems | -.04 | .36 | -.12** | .01 | -.01 | .88 | -.08 | .06 | .06 | .16 |
| Hyperactivity problems | -.30 | .50 | .01 | .82 | -.06 | .15 | -.14* | .001 | -.11* | .01 |
| P-factor | -.03 | .47 | -.02 | .59 | -.07 | .13 | -.11* | .01 | .02 | .66 |

*Note: * = p≤.05; ** = p≤.001*

*Table S7 – Associations between latent variables and EF measures (males only; n=559)*

|  | Working Memory (AWMA) | | Episodic Memory (PSM) | | Flexibility (DCCS) | | Inhibition (Flanker) | | Sustained Attention (Pursuit) | |
| --- | --- | --- | --- | --- | --- | --- | --- | --- | --- | --- |
|  | *r* | *p* | *r* | *p* | *r* | *p* | *r* | *p* | *r* | *p* |
| Latent Variable |  |  |  |  |  |  |  |  |  |  |
| Emotional problems | .17 | .71 | .04 | .38 | -.05 | .31 | .01 | .96 | -.04 | .36 |
| Peer problems | -.10* | .03 | .01 | .88 | -.16** | <.001 | -.03 | .52 | .01 | .95 |
| Conduct problems | -.07 | .14 | -.12* | .01 | -.07 | .14 | -.02 | .75 | .05 | .30 |
| Hyperactivity problems | .06 | .16 | .01 | .92 | -.06 | .20 | -.05 | .27 | .01 | .87 |
| P-factor | -.07 | .10 | -.02 | .63 | -.13* | .01 | -.04 | .45 | .01 | .96 |

*Note: * = p≤.05; ** = p≤.001*

*Table S8 – Associations between latent variables and EF measures (females only; n = 231)*

|  | Working Memory (AWMA) | | Episodic Memory (PSM) | | Flexibility (DCCS) | | Inhibition (Flanker) | | Sustained Attention (Pursuit) | |
| --- | --- | --- | --- | --- | --- | --- | --- | --- | --- | --- |
|  | *r* | *p* | *r* | *p* | *r* | *p* | *r* | *p* | *r* | *p* |
| Latent Variable |  |  |  |  |  |  |  |  |  |  |
| Emotional problems | .06 | .44 | .02 | .81 | .02 | .81 | -.02 | .78 | .21* | .003 |
| Peer problems | -.04 | .62 | -.18* | .01 | -.13 | .06 | -.12 | .11 | .01 | .85 |
| Conduct problems | -.08 | .27 | -.08 | .23 | .03 | .72 | -.23* | .001 | .05 | .52 |
| Hyperactivity problems | -.10 | .14 | -.10 | .14 | -.07 | .30 | -.24** | <.001 | -.24** | <.001 |
| P-factor | -.05 | .50 | -.11 | .12 | -.05 | .50 | -.21* | .003 | .01 | .96 |

*Note: * = p≤.05; ** = p≤.001*
